# Preliminary Screening for *Ophidiomyces ophidiicola* in Pet Snakes from Italy and Exploratory Evaluation of Droplet Digital PCR Assay

**DOI:** 10.3390/microorganisms14020392

**Published:** 2026-02-06

**Authors:** Matteo Riccardo Di Nicola, Simona Sciuto, Daniele Marini, Luca Colla, Giacomo Vanzo, Gabriele Carsana, Emanuele Scanarini, Luana Dell’Atti, Giulia Milanese, Martina Alessandra Gini, Maria Claudia Palazzolo, Jean-Lou C. M. Dorne, Maria Goria, Silvia Colussi, Pier Luigi Acutis

**Affiliations:** 1 Istituto Zooprofilattico Sperimentale del Piemonte, Liguria e Valle d’Aosta, Via Bologna 148, 10154 Turin, Italy; matteoriccardo.dinicola@izsplv.it (M.R.D.N.); luana.dellatti@izsplv.it (L.D.); giulia.milanese@izsplv.it (G.M.); martina.gini@izsplv.it (M.A.G.); maria.palazzolo@izsplv.it (M.C.P.); maria.goria@izsplv.it (M.G.); silvia.colussi@izsplv.it (S.C.); pierluigi.acutis@izsplv.it (P.L.A.); 2 Department of Veterinary Medicine, University of Perugia, Via San Costanzo 4, 06126 Perugia, Italy; 3 Department of Biology, University of Pisa, Via Luca Ghini 13, 56126 Pisa, Italy; luca.colla@phd.unipi.it; 4 Independent Researcher, Via A. Bionda, 28802 Mergozzo, Italy; giacomovanzo@gmail.com; 5 Italian Gekko Association, Via M. Gandhi, 16, 42123 Reggio Emilia, Italy; formazione@italiangekko.net (G.C.); emanuele.scanarini@italiangekko.net (E.S.); 6 Ambulatorio Veterinario Associato Bardolino, Via Colombo 6, 37011 Bardolino, Italy; 7 Methodology and Scientific Support Unit, European Food Safety Authority (EFSA), 43126 Parma, Italy; jean-lou.dorne@efsa.europa.eu

**Keywords:** ddPCR, EIDs, fungal infection, health surveillance, molecular diagnostic assay, pet snakes, SFD, ophidiomycosis

## Abstract

*Ophidiomyces* *ophidiicola*, the agent of ophidiomycosis, has recently been reported in wild snakes in Italy, but the status of captive populations remains unknown. We carried out an opportunistic survey of pet snakes from private collections and, in parallel, performed an exploratory evaluation of a droplet digital PCR (ddPCR) assay adapted from an established probe-based real-time PCR. Non-invasive skin swabs were collected by 32 private owners from 97 snakes, representing 31 species across ten Italian regions. All swabs tested negative for *O. ophidiicola* by both methods, including samples from four snakes that showed cutaneous lesions at the time of sampling. Both assays yielded consistent amplification up to the 1:1000 dilution (ddPCR 0.38 to 0.94 copies/µL for the culture-derived control and 0.24 to 0.33 copies/µL for the field-derived control at 1:1000), while ddPCR retained positive partitions in some replicates at higher dilutions (up to 1:8000). These results provide preliminary screening data for *O. ophidiicola* in an opportunistic sample of Italian pet snakes and suggest potential applicability of ddPCR as a complementary tool for low-template diagnostics, while highlighting the need for larger, standardised surveys and formal assay validation.

## 1. Introduction

*Ophidiomyces ophidiicola*, the aetiological agent of ophidiomycosis, has become a major focus of research on emerging infectious diseases of free-ranging snakes in Europe [1]. This onygenalean fungus primarily infects the keratinised epidermis [2,3,4] and is known to cause infection ranging from mild to severe, potentially becoming life-threatening [2,5,6]. Clinically, *O. ophidiicola* is known to cause swelling, crusts, dermatitis and ulceration, often with dysecdysis and increased shedding; lesions can progress to dermal or hypodermal granulomas and, in disseminated cases, the respiratory tract and other internal organs. However, *O. ophidiicola* can also be detected in snakes without overt clinical signs or visible lesions [7,8]. Behavioural changes related to *O. ophidiicola* presence are also reported [9,10,11].

*Ophidiomyces ophidiicola* was first described in the United States (US) from a case in Georgia as *Chrysosporium ophiodiicola*, and retrospective screening of US museum specimens revealed earlier occurrences dating back to 1945 [12,13,14]. Its presence is now documented in both wild and captive snakes across four continents, including North America, Europe, Asia and Australia [5,8,14,15,16,17,18,19], whereas published detections from South America and Africa are currently lacking, likely reflecting limited surveillance and sampling efforts.

Cultures later reidentified as *O. ophidiicola* indicate that the fungus was present in captive snakes at the latest by 1985 in England (*Python regius*) and by 1986 in the US (*Pantherophis guttatus*) [8,14]. Subsequent captive cases have been reported in Australia, France, Germany, Japan, Russia, the United Kingdom and the US, involving species from at least eight families (Acrochordidae, Boidae, Colubridae sensu lato, Elapidae, Homalopsidae, Natricidae, Pythonidae and Viperidae; Table 1).

Population genomic analyses [32] support multiple introductions of *O. ophidiicola* lineages into North America, plausibly mediated by transcontinental movements of snakes in captive collections, with *O. ophidiicola* potentially residing in the USA for decades before widespread expansion in free-ranging populations. Characterised isolates from captive snakes on three continents belong to Clades II and III [32].

In Italy, *O. ophidiicola* has been detected in museum specimens [33,34], as well as in wild snakes [34,35]. Following its first detection in four free-ranging *Natrix tessellata* from the Garda Lake [35], Di Nicola and colleagues [34] reported a more widespread geographic and taxonomic distribution of the pathogen, detecting *O. ophidiicola* in five of the 22 snake species present in Italy [36]. In addition, a recent survey reported the highest *O. ophidiicola* prevalence recorded for Europe in *N. tessellata*, suggesting Italy as one of the hotspots for the pathogen [11]. Italy is one of the most herpetologically diverse countries in Europe [37] and may therefore be particularly susceptible to further introductions via the pet trade, warranting targeted monitoring of this pathway.

Despite the widespread presence of captive snakes in Italy, the possible endemic presence of the pathogen, and the country’s high biodiversity, no published screening of captive snakes had been conducted prior to this work. Accordingly, this study had two objectives: first, to perform a preliminary, opportunistic screening for *O. ophidiicola* in pet snakes kept by private owners in Italy using non-invasive skin swabs; second, to implement a droplet digital PCR (ddPCR) workflow for *O. ophidiicola* and to undertake a proof-of-concept analytical comparison with a probe-based real-time PCR (qPCR) by testing matched serial dilutions of field- and culture-derived positive controls, thereby assessing detection performance under low-template conditions that may be encountered when swabbing snakes without visible skin lesions. Notably, the ddPCR component and its analytical comparison with qPCR were defined a priori and conducted in parallel, independent of the screening outcome. We hypothesised that ddPCR might retain detection at lower target concentrations than qPCR in this low-template setting, while enabling absolute quantification without the need for standard curves through digital partitioning and potentially offering improved precision and reproducibility [38,39].

## 2. Materials and Methods

### 2.1. O. ophidiicola Detection in Pet Snakes

To investigate the presence of *O. ophidiicola* in pet snakes kept in Italy, private owners voluntarily participated following dissemination of the study through the authors’ networks (convenience sampling). Non-invasive skin swabs were collected by 32 private owners from 97 snakes belonging to 31 species, housed across 10 Italian regions (Table S1).

The swabbing technique suggested to the private keepers followed that of Marini and colleagues [35,40]; sterile dry swabs and written sampling instructions were provided. For each snake, species, country of birth, age class, current region of housing, and the presence of clinical signs compatible with ophidiomycosis were recorded, including dysecdysis, cutaneous swelling, crusted lesions, and poor body condition. Clinical signs were recorded based on owner observation at the time of swabbing. A single sterile dry swab was collected per animal and frozen at −18 °C after collection. Sample logistics were coordinated with the assistance of the Italian Gekko Association, which arranged retrieval and refrigerated shipment to maintain the cold chain, and the material was submitted to the Istituto Zooprofilattico Sperimentale del Piemonte, Liguria e Valle d’Aosta (Turin, Italy), for laboratory analyses. Upon arrival, swabs were stored at −20 °C until DNA extraction, and extracted DNA was stored at −20 °C until molecular testing.

This owner-driven convenience sampling approach was intended as a preliminary exploratory screening and was not designed to estimate prevalence in the overall Italian pet snake population.

DNA from swab samples was extracted with the ReliaPrep gDNA Tissue Miniprep System (Promega, Madison, WI, USA) using the manufacturer’s buccal swab protocol.

The presence of *O. ophidiicola* was investigated across all analysed pet snake samples using both qPCR and ddPCR, each targeting the internal transcribed spacer 2 (ITS2) region of the ribosomal RNA gene complex (primers Oo-rt-ITS-F and Oo-rt-ITS-R were used with the probe Oo-rt-ITS-P from Bohuski et al., 2015 [41]).

The probe-based qPCR assay was previously validated in our system using a five-point 4-fold dilution series. The resulting standard curve exhibited high linearity (R^2^ = 0.999) and an amplification efficiency of 99.8% (see Figure S1). In this qPCR system, each DNA sample from the swabs was tested under two conditions (undiluted and 1:10 diluted) to assess potential inhibition effects, and each condition was run in technical duplicate. Each 20 µL reaction consisted of 10 µL iTaq Universal Probe Supermix (2X; Metabion, Planegg, Germany), 0.8 µL Oo-rt-ITS-F (10 µM) and 0.8 µL Oo-rt-ITS-R (10 µM) (final concentration 400 nM each), 0.4 µL of the Oo-rt-ITS-P probe (10 µM; final concentration 200 nM) [41], 4 µL nuclease-free water, and 4 µL of the DNA template. Thermal cycling was performed on a CFX96™ Touch Real-Time PCR Detection System (Bio-Rad Laboratories Inc., Hercules, CA, USA) with the following cycling conditions: initial denaturation at 95 °C for 3 min, followed by 40 cycles of 95 °C for 3 s and 60 °C for 30 s. Each run included two positive controls (DNA from a confirmed *O. ophidiicola*-positive *N. helvetica* and culture-derived *O. ophidiicola* mycelium), a no-template control (NTC) and an extraction blank. Samples with no amplification or a quantification cycle (Cq) value above 36 were considered negative, following Bohuski et al. (2015) [41]. Acknowledging that cut-offs may vary across different laboratory conditions, we applied this value as a conservative threshold and excluded any later amplification accordingly.

For the ddPCR, *O. ophidiicola* detection was carried out using the same ITS2 primer-probe set as for qPCR (Oo-rt-ITS-F, Oo-rt-ITS-R, and Oo-rt-ITS-P; [41]), adapted to a droplet digital platform. A mix containing 10 µL of 2X ddPCR Supermix for Probes (no dUTP; Bio-Rad), 0.9 µL of forward primer (20 µM), 0.9 µL of reverse primer (20 µM) (final concentration 818 nM each), 1 µL of probe (5 µM; final concentration 227 nM), 5 µL of DNA and 4.2 µL of nuclease-free water was used, for a total volume of 22 µL. A volume of 20.5 µL of the mixture was loaded into a well of a DG8 cartridge and 70 µL of Droplet Generation Oil was added. For droplet generation, the QX200^TM^ Droplet Generator (Bio-Rad) was used, and 40 µL of droplets was transferred into a ddPCR 96-well plate (Bio-Rad). For PCR amplification, the thermal conditions were: denaturation at 95 °C for 10 min, followed by 45 cycles of 94 °C for 30 s, annealing at 58 °C for 1 min and a final step of 98 °C for 10 min. Droplets were analysed by the QX 200 ^TM^ Droplet Reader (Bio-Rad). In order to distinguish between positive and negative samples, a manual threshold was applied by visual inspection of the 1D fluorescence amplitude plots, placing the cut-off between the negative and positive droplet populations using the positive controls as reference, and confirming the absence of positive droplets in NTCs and extraction blanks; the same threshold was then applied to all wells within each run. Results were expressed as copy number/µL, using Quanta Soft ^TM^ (QuantaSoft Analysis Pro v1.0, Bio-Rad). The threshold for the accepted droplets was ≥10,000 per well. A ddPCR result was considered positive when both technical replicates yielded a non-zero concentration estimate. Runs included the same controls described for qPCR.

### 2.2. Relationship Between qPCR and ddPCR Assay

To compare the analytical sensitivity under low-template conditions and the overall analytical performance of qPCR and ddPCR, we tested two confirmed *O. ophidiicola* genomic DNA extracts prepared as controls: (i) field-derived skin tissue from a *Natrix helvetica* and (ii) culture-derived *O. ophidiicola* mycelium. The concentration of double-stranded DNA in the original, undiluted extracts was measured using a Qubit™ dsDNA High Sensitivity assay (Thermo Fisher Scientific, Waltham, MA, USA) and was 2.22 ng/µL for the field-derived extract and 0.115 ng/µL for the culture-derived extract. From the same starting extracts, we prepared matched serial dilutions and tested both assays in duplicate using the primer–probe set and protocols described in Section 2.1.

An initial wide dilution panel was run on each control as undiluted; 1:10; 1:100; 1:1000; 1:10,000; 1:100,000; 1:250,000; and 1:500,000. As neither assay produced positive signals beyond 1:10,000 and both detected up to 1:1000, we then performed a focused second panel, diluting the controls another time, to refine the range around the apparent detection limit: undiluted, 1:10, 1:100, 1:1000, 1:2000, 1:4000, and 1:8000.

To examine the relationship between qPCR and ddPCR, we restricted the analysis to the first dilution panel of each assay and included only dilutions that yielded qPCR amplification with Cq ≤ 36 in both duplicates and non-zero ddPCR concentration estimates in both duplicates. For qPCR, arithmetic means of duplicate reactions showing a clear sigmoidal amplification curve with Cq ≤ 36 were retained. For ddPCR, only dilutions with valid droplet counts and non-zero concentration estimates (copies/µL) were included, expressed as arithmetic means of duplicates. Since the assumption of normality was not met, the relationship between qPCR and ddPCR results was assessed using Spearman’s rank correlation (ρ), after reversing Cq values (−Cq) to account for their inverse relationship with target concentration, acknowledging that correlation reflects association rather than methodological equivalence.

## 3. Results

### 3.1. Screening Outcomes

**Figure 1:**
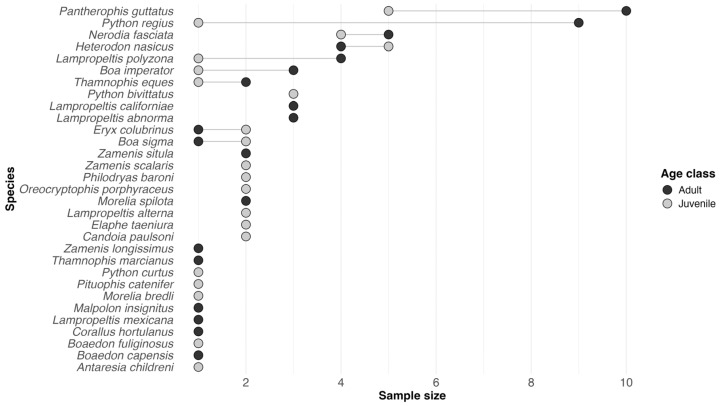
Cleveland dot plot showing the number of screened pet snakes per species and age class.

A total of 97 pet snakes from 31 species were sampled across ten Italian regions (Table S1). The four most represented species were *Pantherophis guttatus* (15.5%), *Python regius* (10.3%), *Nerodia fasciata* (9.3%) and *Heterodon nasicus* (9.3%). Of 94 snakes with known sex, 49 were females (52.1%) and 45 were males (47.9%); age classes comprised 55 adults (56.7%) and 42 juveniles (43.3%) (Figure 1). Most samples originated from Lombardy (22.7%), Sicily (21.6%), and Piedmont (17.5%). Seventy-five snakes were reported as Italy-born (77.3%), whereas 22 were foreign-born (22.7%). Four individuals (4.1%) displayed cutaneous lesions at sampling: two showed cutaneous swellings, one had small, localised lesions on the dorsal scales, and one presented a defect of the left spectacle. However, lesions were owner-reported, not independently assessed by a veterinarian, and were considered non-specific. All skin swabs tested negative for *O. ophidiicola* by both qPCR (neat and 1:10 dilutions) and ddPCR (undiluted) assays. Positive controls were amplified in every run, and no-template controls and extraction blanks were consistently negative.

### 3.2. qPCR and ddPCR Outcomes

For the first wide dilution panel, the culture-derived positive control showed consistent positive detections by both qPCR (i.e., amplification ≤ 36 Cq) and ddPCR up to the 1:1000 dilution. For the field-derived control extracted from skin tissue, both qPCR and ddPCR produced duplicate positive detections up to the 1:100 dilution. At the 1:1000 dilution, ddPCR still detected low copy numbers of *O. ophidiicola* DNA, whereas qPCR reactions were negative according to the Cq > 36 cut-off (Table S2).

In the second dilution panel of the culture-derived control, qPCR showed positive detection in both duplicates up to the 1:100 dilution. For ddPCR, positive results were obtained in both duplicates up to 1:2000, with single positive replicates detected at 1:4000 and 1:8000. For the field-derived DNA extract, both assays yielded amplification in both duplicates up to the 1:100 dilution. A single positive replicate was observed at 1:2000 in ddPCR (Table S3).

Based on the arithmetic means of the amplified duplicates in the first dilution panel, Spearman’s rank correlation between qPCR and ddPCR results revealed a strong and statistically significant positive association between the results of the two methods (ρ = 0.964, *p* = 0.003; Table S3).

## 4. Discussion

In this study, we present an owner-driven, convenience screening of captive pet snakes housed in Italy for *O. ophidiicola*. Non-invasive skin swabs were collected from 97 individuals belonging to 31 species, kept by 32 private owners across ten regions, and all samples tested negative by both qPCR and ddPCR, including those from the few animals that displayed cutaneous lesions at sampling. In addition, we performed a proof-of-concept comparison between qPCR and ddPCR on matched serial dilutions of field- and culture-derived *O. ophidiicola* positive controls, which showed broadly concordant results and a strong positive association between Cq values and ddPCR copy number estimates. At the lowest template concentrations, ddPCR still generated a detectable signal at dilutions where qPCR no longer met our positivity criterion under the conservative Cq cut-off.

Given the opportunistic nature of our sampling design and the limited number of individuals examined, the lack of *O. ophidiicola* detection in pet snakes from Italy cannot be interpreted as evidence for the absence of the pathogen in the wider Italian captive population. Participation was voluntary and swabs were collected by owners following written instructions; therefore, variability in swabbing technique and in the recognition and reporting of skin lesions is possible, and collection history and husbandry variables (including animal source and trade history) were not systematically recorded, precluding assessment of collection-level risk factors. Baseline surveys of this kind remain important in a country where high herpetological diversity [37] intersects with its position within the European Union, one of the major global markets for the live reptile trade [42], and where the pet trade and captive husbandry may act as potential pathways for pathogen movement [2,43].

*Ophidiomyces ophidiicola* has been repeatedly detected in captive snakes worldwide (Table 1), often in species that are widely traded internationally, supporting the view that collections can function both as reservoirs and as hubs of dissemination. In a context where *O. ophidiicola* is already documented in free-ranging snakes in Italy [11,34,35], spillover and co-introduction of this and other snake pathogens from captive to wild populations may represent a potential concern [8,32,44], for example through animal escapes, intentional releases [45,46] or the movement of contaminated substrates and equipment [47,48].

Population genomic analyses further indicate that movements of snakes through the pet trade and other captive pathways have likely contributed to recent, repeated transcontinental translocations of *O. ophidiicola* lineages [32]. In line with this, recent European-scale work supports a role of pathogen clade, together with host identity, in shaping landscape-scale variation in *O. ophidiicola* prevalence and hotspot occurrence [49]. In our dataset, all snakes were sourced via captive pathways; accordingly, additional screening in Italy would be most informative if risk-based, prioritising clinically suspect animals and higher-turnover, mixed-species collections, while recording host identity and key husbandry or enclosure contexts that may influence exposure or environmental persistence (e.g., substrate type or outdoor housing). If *O. ophidiicola* is detected in pet snakes kept in Italy, population genomic typing could then help infer likely sources and potential links with free-ranging Italian cases.

The ddPCR workflow for *O. ophidiicola* was adapted and explored by translating the Bohuski ITS2 TaqMan assay to a partitioned end-point format and we compared it analytically with the established qPCR on matched serial dilutions from field- and culture-derived positives [38,41]. This choice was motivated by the low-template yields and occasional co-extraction of inhibitors typical of non-invasive swabbing of snakes with intact skin, especially in animals without overt lesions [50,51,52]. Under such conditions, digital PCR may improve detection at very low copy numbers and shows greater tolerance to common PCR inhibitors compared with qPCR [38,39,53]. These advantages have also been leveraged in herpetofaunal pathogen surveillance using ddPCR for chytrid fungi in environmental DNA, reinforcing the platform’s suitability for low-template diagnostics [54,55].

Our head-to-head comparison of qPCR and ddPCR under low-template conditions showed broadly similar performance within the concentration range where both assays yielded positive results, with qPCR and ddPCR jointly detecting *O. ophidiicola* DNA up to a 1:1000 dilution for the culture-derived control and up to 1:100 for the field-derived control. Notably, at the 1:1000 dilution of the field-derived control, ddPCR remained positive in both technical replicates, while qPCR produced late amplification in both technical replicates (Cq 38.51 and 39.31; Table S2); these qPCR values exceeded our conservative Cq 36 threshold and were therefore considered negative, while still warranting consideration as signals near the detection limit. At lower template concentrations, however, ddPCR showed indications of higher operational sensitivity in this set-up, with both technical replicates remaining positive at 1:2000 for the culture-derived control, sporadic positive partitions at higher dilutions (single replicates at 1:4000 and 1:8000), and a single positive replicate at 1:2000 for the field-derived control, whereas qPCR results at these dilutions were considered negative under the conservative Cq > 36 cut-off. Single-replicate detections at these highest dilutions were treated as exploratory near-limit signals and were not interpreted as evidence of reliable or consistent detection. The positive correlation observed between Cq values and ddPCR copy number estimates across this overlapping positive range supports the association between the two methods and suggests overall concordance in their analytical performance. Nevertheless, this does not demonstrate methodological equivalence and should instead be regarded as an initial proof-of-concept. Accordingly, our assessment was designed as an exploratory analytical comparison of detection performance under low-template, field-relevant conditions rather than a formal validation, consistent with Minimum Information for Publication of Quantitative Digital PCR Experiments (dMIQE) guidance on reporting and interpretation [38].

Furthermore, larger scale surveys of pet snakes in Italy will be essential to better characterise *O. ophidiicola* occurrence in Italian captive snakes, and, where present, to quantify prevalence at the level of collections and host species. Moreover, our results suggest that the ddPCR assay explored in this study may be a potentially useful adjunct for *O. ophidiicola* detection from low-template, non-invasive samples, pending formal validation.

## Figures and Tables

**Table 1:** Published *O. ophidiicola* detection in captive snakes across the world.

Reference	Species	Country
[20] *	*Boiga irregularis*	United States
[21] *	*Thamnophis* sp.	Germany
[22] *	*Python sebae*	United States
[23] *	*Eunectes murinus*	United States
[13] *	*Pantherophis alleghaniensis*	United States
[24] *	*Hoplocephalus bungaroides*	Australia
[14]	*Pantherophis guttatus*, *Lampropeltis* sp., *Nerodia clarkii*, *Python regius*, *Acrochordus* sp.	United States, United Kingdom, Australia
[5]	*Agkistrodon piscivorous*	United States
[25]	*Coluber constrictor*, *Thamnophis sirtalis*	United States
[26]	*Sistrurus catenatus*	United States
[27]	*Subsessor bocourti*, *Lampropeltis triangulum*	France
[28]	*Eunectes murinus*, *E. notaeus*, *Corallus hortolanus*, *Hydrodynastes gigas*, *Lampropeltis triangulum*, *Epicrates cenchria*, *Crotalus adamanteus*	United States
[29]	*Acrochordus granulatus*	Russia
[30]	*Lampropeltis californiae*, *Lampropeltis getula*, *Pantherophis guttatus*	United States
[16]	*Pantherophis obsoletus*	Japan
[31]	*Eunectes murinus*	France

* Initially assigned to other Onygenales; later reclassified as *O. ophidiicola*.

## Data Availability

The original contributions presented in this study are included in the article/Supplementary Materials. Further inquiries can be directed to the corresponding authors.

## Supplementary Materials

The following supporting information can be downloaded at: https://www.mdpi.com/article/10.3390/microorganisms14020392/s1, Table S1. List of snakes screened for *Ophidiomyces ophidiicola* by qPCR and ddPCR. Species names follow Uetz et al., 2025 [56]. The Housing region column refers to where the animal is currently housed in Italy. Table S2. Amplification outcomes of qPCR and ddPCR for *Ophidiomyces ophidiicola* across matched serial dilutions of field- and culture-derived positive controls. ‘N/P’ means not performed; ‘/’ indicates no amplification. Cells highlighted in yellow show qPCR amplification with a Cq greater than 36. Table S3. qPCR and ddPCR results for the subset of culture- and field-derived *Ophidiomyces ophidiicola*-positive controls included in the Spearman rank correlation analysis. ‘BIS’ refers to a technical replicate. Cells highlighted in yellow show qPCR amplification with a Cq greater than 36. Figure S1. Efficiency curve of the probe-based qPCR assay targeting the *Ophidiomyces ophidiicola* ITS2 region, originally from Bohuski et al., 2015 [41]. The assay was validated using a five-point, four-fold serial dilution of positive control DNA (extracted from culture). The standard curve showed linearity of 0.9997 (R^2^) and an amplification efficiency of 99.8% (E = 10^(–1/a)^ – 1 = 0.998, calculated from the slope of −3.325). Average Cq values (±SD) for the dilution series were 25.48 (±0.13), 27.38 (±0.03), 29.35 (±0.37), 31.41 (±0.16), and 33.47 (±0.28), respectively.
