# Supplementary material for: Preliminary Screening for Ophidiomyces ophidiicola in Pet Snakes from Italy and Exploratory Evaluation of Droplet Digital PCR Assay
Source: Microorganisms. 2026 Feb 6;14(2):392. doi: 10.3390/microorganisms14020392 (PMC12943615; doi:10.3390/microorganisms14020392)

Preliminary Screening for *Ophidiomyces ophidiicola* in Pet Snakes from Italy and Exploratory Evaluation of a Droplet Digital PCR Assay

Matteo Riccardo Di Nicola, Simona Sciuto, Daniele Marini, Luca Colla, Giacomo Vanzo, Gabriele Carsana, Emanuele Scanarini, Luana Dell'Atti, Giulia Milanese, Martina Alessandra Gini, Maria Claudia Palazzolo, Jean-Lou C. M. Dorne, Maria Goria, Silvia Colussi, and Pier Luigi Acutis

SUPPLEMENTARY MATERIAL

Table S1: List of snakes screened for *Ophidiomyces ophidiicola* by qPCR and ddPCR. Species names follow Uetz et al., 2025 [56]. The Housing region column refers to where the animal is currently housed in Italy.

Sample ID	Species	Sex	Age class	Housing region	Country of Birth	Skin lesions
1	*Antaresia childreni*	Male	Juvenile	Lazio	Czech Republic	No
2	*Boa imperator*	Male	Adult	Trentino-South Tyrol	Italy	Yes
3	*Boa imperator*	Male	Juvenile	Lombardy	Slovenia	No
4	*Boa imperator*	Male	Adult	Emilia-Romagna	Hungary	No
5	*Boa imperator*	Female	Adult	Sicily	Italy	No
6	*Boa sigma*	Female	Juvenile	Friuli-Venezia Giulia	Italy	No
7	*Boa sigma*	Female	Adult	Piedmont	Italy	No
8	*Boa sigma*	N/A	Juvenile	Lazio	France	No
9	*Boaedon capensis*	Male	Adult	Lombardy	Italy	No
10	*Boaedon fuliginosus*	Female	Juvenile	Sicily	Italy	No
11	*Candoia paulsoni*	Male	Juvenile	Piedmont	Bulgaria	No
12	*Candoia paulsoni*	Female	Juvenile	Piedmont	Italy	No
13	*Corallus hortulanus*	Male	Adult	Friuli-Venezia Giulia	Italy	No
14	*Elaphe taeniura*	Male	Juvenile	Lazio	Germany	No
15	*Elaphe taeniura*	Female	Juvenile	Lazio	Germany	No
16	*Eryx colubrinus*	Male	Juvenile	Lazio	Netherlands	No
17	*Eryx colubrinus*	Male	Juvenile	Lazio	Netherlands	No
18	*Eryx colubrinus*	Female	Adult	Lombardy	Italy	No
19	*Heterodon nasicus*	Male	Juvenile	Lazio	Italy	No
20	*Heterodon nasicus*	Female	Juvenile	Lazio	Spain	No
21	*Heterodon nasicus*	Female	Juvenile	Lazio	Italy	No
22	*Heterodon nasicus*	Male	Adult	Piedmont	Italy	No
23	*Heterodon nasicus*	Male	Adult	Piedmont	Italy	No
24	*Heterodon nasicus*	Female	Adult	Piedmont	Italy	No
25	*Heterodon nasicus*	Female	Juvenile	Piedmont	Italy	No
26	*Heterodon nasicus*	Female	Juvenile	Piedmont	Italy	No
27	*Heterodon nasicus*	Female	Adult	Sicily	Italy	Yes
28	*Lampropeltis abnorma*	Male	Adult	Emilia-Romagna	Italy	No

29	*Lampropeltis abnorma*	Female	Adult	Emilia-Romagna	Czech Republic	No
30	*Lampropeltis abnorma*	Male	Adult	Sicily	Italy	No
31	*Lampropeltis alterna*	Female	Juvenile	Piedmont	Italy	No
32	*Lampropeltis alterna*	Female	Juvenile	Piedmont	Italy	No
33	*Lampropeltis californiae*	Male	Adult	Sicily	Italy	No
34	*Lampropeltis californiae*	Female	Adult	Piedmont	Italy	No
35	*Lampropeltis californiae*	Female	Adult	Lombardy	Italy	No
36	*Lampropeltis mexicana*	Male	Adult	Lazio	Italy	No
37	*Lampropeltis polyzona*	Female	Adult	Tuscany	Czech Republic	No
38	*Lampropeltis polyzona*	Male	Juvenile	Emilia-Romagna	Slovakia	No
39	*Lampropeltis polyzona*	Female	Adult	Sicily	Italy	No
40	*Lampropeltis polyzona*	Male	Adult	Veneto	Bulgaria	No
41	*Lampropeltis polyzona*	Female	Adult	Veneto	Germany	No
42	*Malpolon insignitus*	Male	Adult	Trentino-South Tyrol	Italy	No
43	*Morelia bredli*	Female	Juvenile	Lombardy	Italy	No
44	*Morelia spilota*	Male	Adult	Sicily	Poland	No
45	*Morelia spilota*	Female	Adult	Sicily	Poland	No
46	*Nerodia fasciata*	Female	Juvenile	Lombardy	Italy	No
47	*Nerodia fasciata*	Female	Juvenile	Lombardy	Italy	No
48	*Nerodia fasciata*	Male	Juvenile	Lombardy	Italy	No
49	*Nerodia fasciata*	Male	Juvenile	Lombardy	Italy	No
50	*Nerodia fasciata*	Female	Adult	Trentino-South Tyrol	Italy	No
51	*Nerodia fasciata*	Female	Adult	Trentino-South Tyrol	Italy	No
52	*Nerodia fasciata*	Female	Adult	Trentino-South Tyrol	Italy	No
53	*Nerodia fasciata*	Male	Adult	Trentino-South Tyrol	Italy	No
54	*Nerodia fasciata*	Female	Adult	Trentino-South Tyrol	Italy	No
55	*Oreocryptophis porphyraceus*	Female	Juvenile	Lazio	Germany	Yes

56	*Oreocryptophis porphyraceus*	Female	Juvenile	Lazio	Germany	No
57	*Pantherophis guttatus*	Female	Adult	Lombardy	Italy	No
58	*Pantherophis guttatus*	Female	Adult	Sicily	Italy	No
59	*Pantherophis guttatus*	Male	Juvenile	Sicily	Italy	No
60	*Pantherophis guttatus*	Female	Juvenile	Sicily	Italy	No
61	*Pantherophis guttatus*	Male	Adult	Sicily	Italy	No
62	*Pantherophis guttatus*	Male	Adult	Lombardy	Italy	No
63	*Pantherophis guttatus*	Male	Juvenile	Lombardy	Italy	No
64	*Pantherophis guttatus*	Male	Juvenile	Lazio	United Kingdom	No
65	*Pantherophis guttatus*	Male	Juvenile	Lazio	Italy	No
66	*Pantherophis guttatus*	Male	Adult	Lombardy	Italy	No
67	*Pantherophis guttatus*	Female	Adult	Lazio	Italy	No
68	*Pantherophis guttatus*	Female	Adult	Calabria	Italy	No
69	*Pantherophis guttatus*	Female	Adult	Sicily	Italy	No
70	*Pantherophis guttatus*	Male	Adult	Veneto	Italy	No
71	*Pantherophis guttatus*	Male	Adult	Lombardy	Italy	No
72	*Philodryas baroni*	Female	Juvenile	Piedmont	Poland	No
73	*Philodryas baroni*	Male	Juvenile	Piedmont	Poland	No
74	*Pituophis catenifer*	Male	Juvenile	Lombardy	Italy	No
75	*Python bivittatus*	Female	Juvenile	Sicily	Italy	No
76	*Python bivittatus*	Male	Juvenile	Sicily	Italy	No
77	*Python bivittatus*	Male	Juvenile	Lombardy	Italy	No
78	*Python curtus*	N/A	Juvenile	Sicily	Italy	No
79	*Python regius*	Male	Juvenile	Lombardy	Italy	No
80	*Python regius*	N/A	Adult	Veneto	Italy	No
81	*Python regius*	Female	Adult	Sicily	Italy	No
82	*Python regius*	Male	Adult	Sicily	Italy	No
83	*Python regius*	Female	Adult	Sicily	Italy	No
84	*Python regius*	Female	Adult	Sicily	Italy	No
85	*Python regius*	Male	Adult	Sicily	Italy	No
86	*Python regius*	Male	Adult	Lombardy	Italy	No

87	*Python regius*	Female	Adult	Lombardy	Italy	No
88	*Python regius*	Female	Adult	Lombardy	Italy	No
89	*Thamnophis eques*	Female	Juvenile	Lombardy	Italy	No
90	*Thamnophis eques*	Female	Adult	Trentino-South Tyrol	Italy	No
91	*Thamnophis eques*	Male	Adult	Trentino-South Tyrol	Italy	Yes
92	*Thamnophis marcianus*	Female	Adult	Lombardy	Italy	No
93	*Zamenis longissimus*	Male	Adult	Trentino-South Tyrol	Italy	No
94	*Zamenis scalaris*	Male	Juvenile	Piedmont	Italy	No
95	*Zamenis scalaris*	Female	Juvenile	Piedmont	Italy	No
96	*Zamenis situla*	Female	Adult	Piedmont	Italy	No
97	*Zamenis situla*	Male	Adult	Piedmont	Italy	No

Table S2. Amplification outcomes of qPCR and ddPCR for *Ophidiomyces ophidiicola* across matched serial dilutions of field- and culture-derived positive controls. 'N/P' means not performed; '/' indicates no amplification. Cells highlighted in yellow show qPCR amplification with a Cq greater than 36.

Oo sample (Dilution)	qPCR 1st run 1st duplicate (Cq)	qPCR 1st run 2nd duplicate (Cq)	qPCR 2nd run 1st duplicate (Cq)	qPCR 2nd run 2nd duplicate (Cq)	ddPCR 1st run 1st duplicate (copies/μL)	ddPCR 1st run 2nd duplicate (copies/μL)	ddPCR 2nd run 1st duplicate (copies/μL)	ddPCR 2nd run 2nd duplicate (copies/μL)
Culture 1:1	25.55	25.29	25.97	26.05	321	335	396	425
Culture 1:10	28.98	28.96	29.57	29.42	33.2	25.1	36.1	34.4
Culture 1:100	32.33	32.34	33.48	33.71	1.51	1.91	4.61	3.81
Culture 1:1,000	35.54	34.48	/	/	0.38	0.94	0.328	1.37
Culture 1:2,000	N/P	N/P	/	/	N/P	N/P	0.315	0.435
Culture 1:4,000	N/P	N/P	/	/	N/P	N/P	/	0.197
Culture 1:8,000	N/P	N/P	37.45	/	N/P	N/P	0.174	/
Culture 1:10,000	/	36.42	N/P	N/P	/	/	N/P	N/P
Culture 1:25,000	/	/	N/P	N/P	/	/	N/P	N/P
Culture 1:50,000	/	/	N/P	N/P	/	/	N/P	N/P
Culture 1:100,000	/	/	N/P	N/P	/	/	N/P	N/P
Natrix 1:1	26.53	26.67	27.05	27.19	142	168	180	173
Natrix 1:10	29.95	29.8	30.41	30.33	13.1	13.7	18.5	17
Natrix 1:100	33.61	34.43	34.93	34.78	2.47	1.7	2.15	1.59
Natrix 1:1,000	38.51	39.31	/	/	0.24	0.33	/	/
Natrix 1:2,000	N/P	N/P	/	/	N/P	N/P	/	0.29
Natrix 1:4,000	N/P	N/P	/	38.7	N/P	N/P	/	/
Natrix 1:8,000	N/P	N/P	/	/	N/P	N/P	/	/
Natrix 1:10,000	/	/	N/P	N/P	/	/	N/P	N/P

Natrix 1:25,000	/	/	N/P	N/P	/	/	N/P	N/P
Natrix 1:50,000	/	/	N/P	N/P	/	/	N/P	N/P
Natrix 1:100,000	/	/	N/P	N/P	/	/	N/P	N/P

Table S3. qPCR and ddPCR results for the subset of culture- and field-derived *Ophidiomyces ophidiicola*-positive controls included in the Spearman rank correlation analysis. 'BIS' refers to a technical replicate. Cells highlighted in yellow show qPCR amplification with a Cq greater than 36.

Oo sample (Dilution)	qPCR 1° run 1st duplicate (Cq)	qPCR 1° run 2nd duplicate (Cq)	ddPCR 1° run 1st duplicate (copies/μL)	ddPCR 1° run 2nd duplicate (copies/μL)	qPCR MEAN (Cq)	ddPCR MEAN (copies/μL)	Negative qPCR MEAN (-Cq)
Culture 1:1	25.55	25.29	321	335	25.42	328	-25.42
Culture 1:10	28.98	28.96	33.2	25.1	28.97	29.15	-28.97
Culture 1:100	32.33	32.34	1.51	1.91	32.335	1.71	-32.335
Culture 1:1,000	35.54	34.48	0.38	0.94	35.01	0.66	-35.01
Natrix 1:1	26.53	26.67	142	168	26.6	155	-26.6
Natrix 1:10	29.95	29.8	13.1	13.7	29.875	13.4	-29.875
Natrix 1:100	33.61	34.43	2.47	1.7	34.02	2.085	-34.02

Figure S1. Efficiency curve of the probe-based qPCR assay targeting the *Ophidiomyces ophidiicola* ITS2 region, originally from Bohuski et al., 2015 [41]. The assay was validated using a five-point, four-fold serial dilution of positive control DNA (extracted from culture). The standard curve showed linearity of 0.9997 (R^2) and an amplification efficiency of 99.8% (E = 10(–1/a) – 1 = 0.998, calculated from the slope of −3.325). Average Cq values (±SD) for the dilution series were 25.48 (±0.13), 27.38 (±0.03), 29.35 (±0.37), 31.41 (±0.16), and 33.47 (±0.28), respectively.

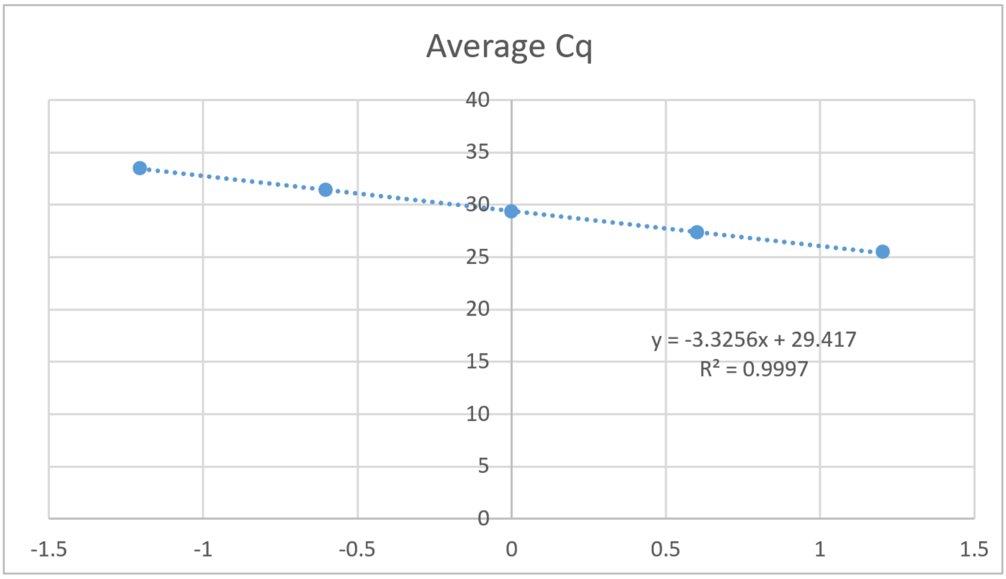

Supplement: Supplementary file 1 [file microorganisms-14-00392-s001.zip › microorganisms-4122909-supplementary.pdf]
